# Nonsteroidal Anti-Inflammatory Drug Conjugated with Gadolinium (III) Complex as an Anti-Inflammatory MRI Agent

**DOI:** 10.3390/ijms24076870

**Published:** 2023-04-06

**Authors:** Bokyung Sung, Hee-Kyung Kim, Ah-Rum Baek, Byeong-Woo Yang, Yeoun-Hee Kim, Garam Choi, Hyun-Jin Park, Minsup Kim, Jongmin Lee, Yongmin Chang

**Affiliations:** 1 Department of Medical & Biological Engineering, Kyungpook National University, Jung-gu, Daegu 41944, Republic of Korea; priere4@gmail.com (B.S.);; 2 Preclinical Research Center, Daegu-Gyeongbuk Medical Innovation Foundation (K-MEDI hub), Dong-gu, Daegu 41061, Republic of Korea; hkkim@kmedihub.re.kr; 3 Institute of Biomedical Engineering Research, Kyungpook National University, Jung-gu, Daegu 41566, Republic of Korea; 4 R&D Center, Etnova Therapeutics Corp., Gwonseon-gu, Suwon-si 13120, Republic of Korea; 5 Department of Biotechnology and Bioinformatics, Korea University Sejong Campus, 2511 Sejong-ro, Sejong City 30019, Republic of Korea; 6 Department of Radiology, Kyungpook National University Hospital, Jung-gu, Daegu 41944, Republic of Korea; 7 Department of Molecular Medicine, School of Medicine, Kyungpook National University, Jung-gu, Daegu 41944, Republic of Korea

**Keywords:** magnetic resonance imaging, contrast agent, NSAIDs, COX-2, anti-inflammation

## Abstract

Studies have been actively conducted to ensure that gadolinium-based contrast agents for magnetic resonance imaging (MRI) are accompanied by various biological functions. A new example is the anti-inflammatory theragnostic MRI agent to target inflammatory mediators for imaging diagnosis and to treat inflammatory diseases simultaneously. We designed, synthesized, and characterized a Gd complex of 1,4,7-tris(carboxymethylaza) cyclododecane-10-azaacetylamide (DO3A) conjugated with a nonsteroidal anti-inflammatory drug (NSAID) that exerts the innate therapeutic effect of NSAIDs and is also applicable in MRI diagnostics. Gd-DO3A-fen (0.1 mmol/kg) was intravenously injected into the turpentine oil-induced mouse model, with Gd-DO3A-BT as a control group. In the in vivo MRI experiment, the contrast-to-noise ratio (CNR) was higher and persisted longer than that with Gd-DO3A-BT; specifically, the CNR difference was almost five times at 2 h after injection. Gd-DO3A-fen had a binding affinity (*K*_a_) of 6.68 × 10^6^ M^−1^ for the COX-2 enzyme, which was 2.1-fold higher than that of fenbufen, the original NSAID. In vivo evaluation of anti-inflammatory activity was performed in two animal models. In the turpentine oil-induced model, the mRNA expression levels of inflammatory parameters such as COX-2, TNF-α, IL-1β, and IL-6 were reduced, and in the carrageenan-induced edema model, swelling was suppressed by 72% and there was a 2.88-fold inhibition compared with the saline group. Correlation analysis between in vitro, in silico, and in vivo studies revealed that Gd-DO3A-fen acts as an anti-inflammatory theragnostic agent by directly binding to COX-2.

## 1. Introduction

Inflammation is a general defense mechanism activated in response to tissue injury and implicated in conditions such as arthritis, neurodegenerative disease, and cancer [1,2,3,4,5,6]. Inflammatory diseases are induced by intricate interactions between impaired tissues and inflammatory cells, which release inflammatory mediators such as interleukins, necrotic factors, and cyclooxygenase (COX) enzymes. Classical nonsteroidal anti-inflammatory drugs (NSAIDs) directly inhibit both COX enzymes (COX-1 and COX-2) [7,8]. COX-1 is constitutively involved in basic housekeeping functions in the body. COX-2 is induced under inflammatory conditions and plays a critical role in the production of prostaglandins (PGs), which are the mediators of inflammation [9]. Because overexpressed COX-2 plays a vital role in the inflammatory pathway [10], inflammation is effectively treated by NSAIDs, which are the most commonly used medications for achieving analgesic, antipyretic, and anti-inflammatory effects [11,12]. Furthermore, due to the correlation between COX-2 upregulation and various pathologies, COX-2 inhibitors have recently been developed as potential agents for targeted imaging [13,14] or therapy [15,16].

NSAIDs can be classified on the basis of their chemical structures as salicylates, arylalkanoic acids, 2-arylpropionic acids (profens), *N*-arylanthranilic acids (fenamic acids), pyrazolidine derivatives, oxicams, sulfonanilides, and others [17,18]. These categories of agents exhibit different degrees of specificity for COX-2 inhibition. Traditional NSAIDs, such as ibuprofen and fenbufen, are nonselective blockers of both the COX isoforms, whereas agents such as celecoxib and rofecoxib are diarylheterocyclic compounds possessing —SO_2_ pharmacophores, which strongly and selectively bind to and inhibit COX-2 enzymes [19,20]. We are already aware that COX-1 inhibition by NSAIDs can cause gastrointestinal side effects, and selective COX-2 inhibitors can cause cardiovascular problems [21]. Nevertheless, NSAIDs are widely prescribed in consideration of the patient’s history as pain relievers or anti-inflammatory drugs by virtue of their positive drug effectiveness.

Moreover, there have been several attempts to realize targeted imaging of inflammation using inflammatory mediators as imaging biomarkers [14,22,23,24,25,26,27]. Of these targeted imaging techniques, magnetic resonance imaging (MRI) has demonstrated several advantages to investigate the human anatomy and physiology with superior spatial resolution. Therefore, a strong motivation exists to expand the scope of gadolinium-based contrast agents (GBCAs) for MRI to design theragnostic agents that not only deliver CAs to the disease site. but can also inhibit inflammatory mediators. In our previous research, we introduced a new type of MRI agent that could suppress COX-2 to prevent reperfusion injury after stroke [28] and NSAIDs demonstrated sufficient potential to develop theragnostic agents for advanced medical technologies.

In the present study, we designed and synthesized a Gd complex (Gd-DO3A-fen) conjugated with a traditional NSAID, fenbufen. Gd-DO3A-fen obviously maintained the therapeutic effect of the anti-inflammatory drug component and diagnosed inflammatory lesions on MRI. A molecular docking study showed that Gd-DO3A-fen has enhanced COX-2 inhibition efficacy by possessing a greater number of binding sites and higher binding energies than fenbufen. This novel agent may help advance the field of theragnostics toward an era of more effective therapeutic approaches.

## 2. Results

### 2.1. Relativities and Kinetic Stability of Gd-DO3A-fen

**Figure 1:**
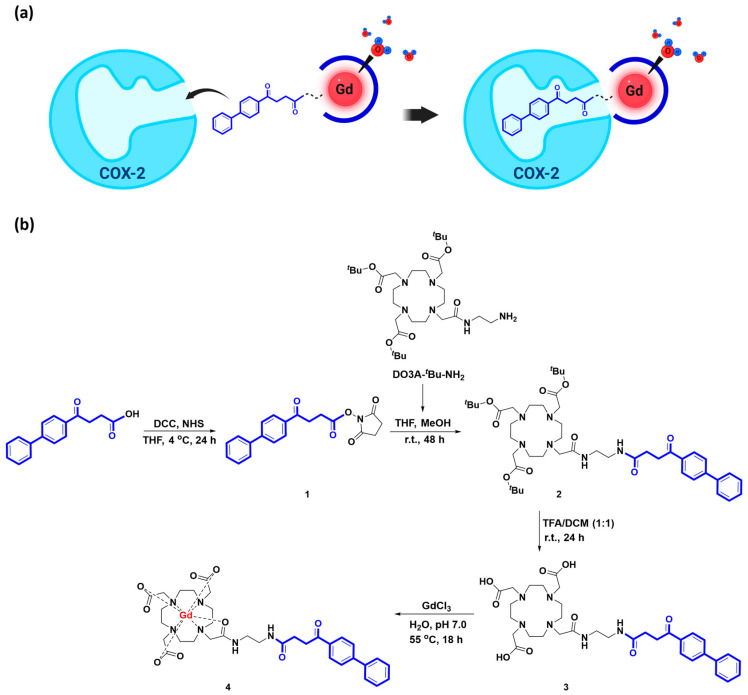
Diagram of Gd-DO3A-fen. (**a**) Representation of the mechanism of action of Gd-DO3A-fen. (**b**) The synthetic procedure of the anti-inflammatory MRI CAs Gd-DO3A-fen.

Gd-DO3A-fen, which conjugated gadolinium chelate with fenbufen, was designed to target COX-2 enzymes in inflammatory lesions (Figure 1a). The synthetic procedure of Gd-DO3A-fen is illustrated in Figure 1b. The *r*_1_ and *r*_2_ relativities of Gd-DO3A-fen were 5.21 ± 0.14 and 5.73 ± 0.12 mM^−1^s^−1^ in PBS solution (pH 7.4), respectively. The *r*_1_ and *r*_2_ relativities of the clinically used GBCA Gd-DO3A-BT were 4.54 ± 0.14 and 4.95 ± 0.14 mM^−1^s^−1^ in PBS solution (pH 7.4), respectively. Gd-DO3A-fen showed slightly higher relativities in PBS than Gd-DO3A-BT (Supplementary Table S1). To simulate physiological conditions as closely as possible, the relativities were measured in a solution of 4.5% human serum albumin (HSA), the most abundant protein in plasma. The relativities of Gd-DO3A-fen increased in the HSA solution, indicating the significant role of the aromatic rings of conjugated fenbufen in lipophilic interactions with HSA (Supplementary Table S1). The lipophilicity of Gd compounds was estimated using the octanol–water partition coefficient Log *P*_oct/wat_, which revealed a higher lipophilicity for Gd-DO3A-fen than for the commercial GBCA Gd-DO3A-BT (Supplementary Table S1).

The kinetic stability of Gd-DO3A-fen was expressed as *R*_1_^P^ (t)/*R*_1_^P^ (0), with normalized paramagnetic longitudinal relaxation rates, compared with the values of Gd-DO3A-BT, Gd-DOTA, Gd-DTPA-EOB, and Gd-DTPA-BOPTA (Supplementary Figure S4). The coordination of Gd^3+^ can be replaced by endogenous ions, particularly Zn^2+^, which is present in human blood at high concentrations (55–125 mM) and has a binding constant suitable for the coordination of Gd chelates [29]. By measuring the change of *R*_1_^P^ values, the transmetalation of Gd^3+^ and Zn^2+^ can be confirmed. Moreover, kinetic stability is somewhat predicted depending by the macrocyclic or linear structure. As we anticipated, Gd-DO3A-fen exhibited high values of kinetic inertness comparable with those of Gd-DO3A-BT using the same structure of macrocyclic chelate. No significant changes were observed in the relaxation rate for 3 days. In contrast, other GBCAs possessing linear DTPA analogs showed a significant decrease in *R*_1_ in the same period.

### 2.2. In Vivo MRI and Biodistribution of Gd-DO3A-fen

**Figure 2:**
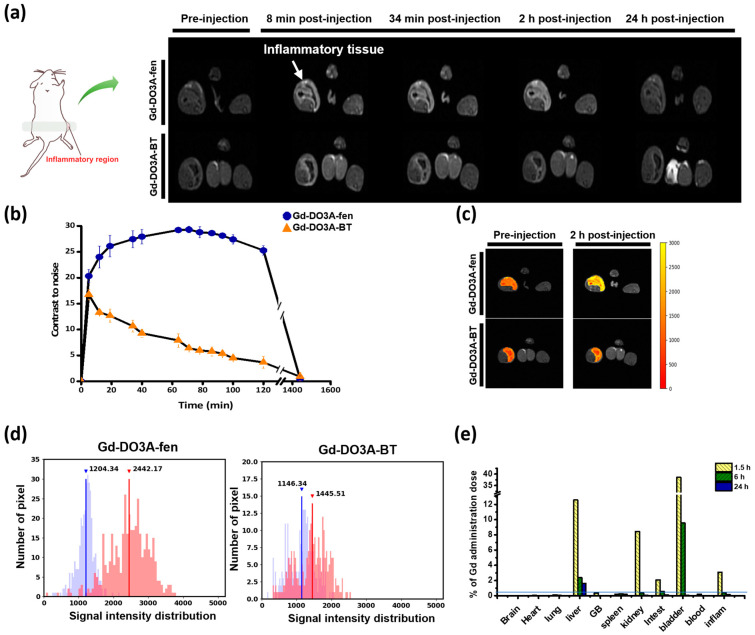
In vivo assessment in the inflammation-induced mouse model. (**a**–**c**) *T_1_*-weighted MR images were acquired before and after the administration of Gd-DO3A-fen and Gd-DO3A-BT (0.1 mmol/kg) by 1.5-T MRI. (**a**) Anatomical axial images of the inflammation site are displayed in gray scale and (**b**) CNR profile is shown as a function of time. Values are expressed as mean ± S.D. (*n* = 3). (**c**) Color-map at maximum-enhanced time. (**d**) Histogram of signal intensity values in the inflammatory region. The blue bars represent the signal intensity distribution for preinjection data, and the red bars represent the data obtained at 2 h postinjection of Gd-DO3A-fen (left) and Gd-DO3A-BT (right). Each line and value on the graph represent the average values for each distribution. (**e**) The inflammation-induced mouse model was sacrificed after the administration of Gd-DO3A-fen to quantify the in vivo biodistribution at each time point (*n* = 4). The results show the amount of Gd (mmol) in each tissue as a percentage (%) of total Gd administration dose (0.1 mmol/kg). The amount of Gd (mmol) in different tissues was determined by ICP-AES. GB, gallbladder; intest, intestines; inflam, inflamed tissue.

*T*_1_-weighted MR images of an inflammation-induced mouse model were obtained before and after intravenous injection of 0.1 mmol/kg Gd-DO3A-fen, with administration of the commercially used extracellular fluid (ECF) agent Gd-DO3A-BT for comparison. We observed increased signal intensity in the inflamed left thigh within 10 min in all Gd chelates, and the strong enhancement of Gd-DO3A-fen lasted as long as 2 h postinjection (Figure 2a). The contrast-to-noise ratio (CNR) of Gd-DO3A-fen was higher and persisted longer in mice than that induced by Gd-DO3A-BT (Figure 2b). The *T*_1_ color map of the inflamed tissue obtained at the peak time of enhancement is shown in Figure 2c. The signal intensities of inflamed tissues injected with Gd-DO3A-fen were increased by more than two times relative to preinjection values. The signal intensities (SI) are represented by the histogram peaks in Figure 2d. The histogram represents a distribution of SI corresponding to the color-mapped area of the inflamed tissue. The red and blue distributions indicate the SI distribution of the inflamed tissue before and after the injection of Gd-DO3A-fen and Gd-DO3A-BT, respectively. Strong and prolonged enhancement in inflamed tissues is considered a consequence of COX-2 targeting by Gd-DO3A-fen.

Whole body MRI biodistribution of Gd-DO3A-fen demonstrated its excretion through both the kidney and gallbladder (Supplementary Figure S5). The hepatobiliary excretion was quantitatively estimated from the measurements of Gd concentration obtained by inductively coupled plasma atomic emission spectrometry (ICP-AES). The partial excretion through the gallbladder was a consequence of hepatocyte uptake in the liver (Figure 2e). Gd concentrations in the inflamed tissue of Gd-DO3A-fen-treated mice revealed a high accumulation of 3% at 1.5 h postinjection. Therefore, it was clearly demonstrated that Gd-DO3A-fen can specifically target inflammatory factors such as COX-2 in the in vivo model.

### 2.3. COX-2-Binding Affinity of Gd-DO3A-fen

**Figure 3:**
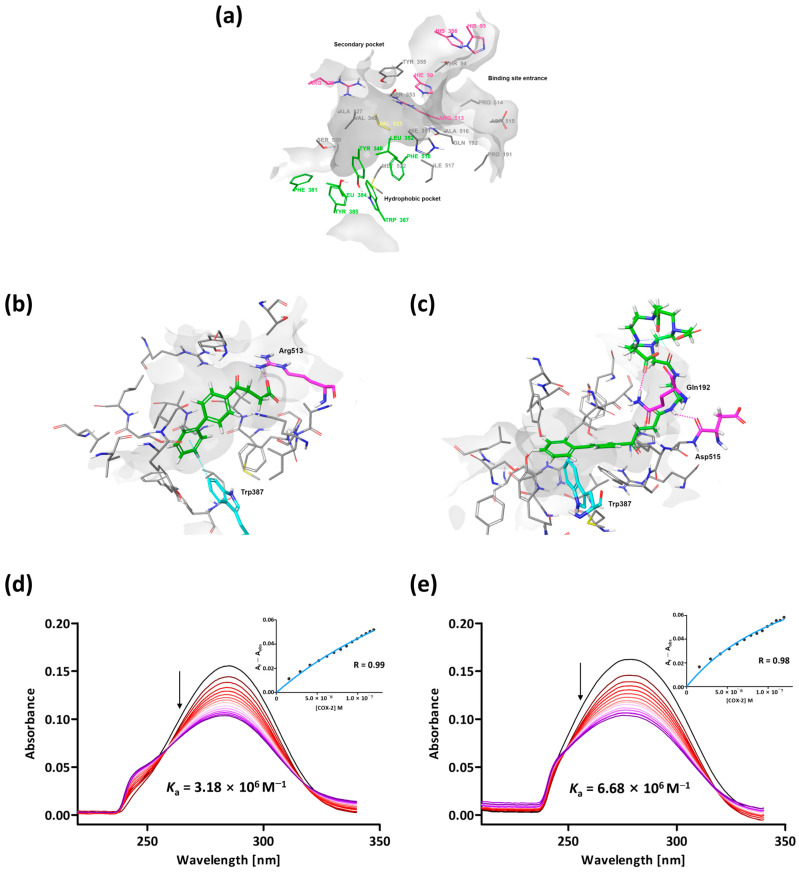
The binding sites and binding affinity of COX-2 for Gd-DO3A-fen and the NSAID. (**a**) The drug-binding site of COX-2 consists of the main hydrophobic and secondary pockets. (**b**) The docking results for fenbufen. This drug was bound to Arg 513, and Trp 387 deep in the hydrophobic pocket, with ionic and π–π stacking interactions. (**c**) The docking results for Gd-DO3A-fen. The binding conformation of Gd-DO3A-fen was associated with polar interactions with surface protein and π–π stacking interactions in the hydrophobic pocket. (**d**) COX-2-binding affinity of fenbufen in UV–vis spectra. Samples were prepared in DMSO. COX-2 was dissolved in Tris-HCl buffer. (**e**) COX-2-binding affinity of Gd-DO3A-fen in UV–vis spectra. Samples were prepared in Tris-HCl (1:9, pH 7.0) buffer. (**d**,**e**) The absorbance of fenbufen and Gd-DO3A-fen were decreased by the addition of COX-2 enzyme. The inset graphs represent the fitting curve for the calculation of the binding constant (*K*_a_).

Protein–ligand docking simulations were performed to reveal the underlying mechanism of the target effect on the inflammatory site of Gd-DO3A-fen that was revealed by the MRI in vivo study. The binding energy between COX-2 and Gd-DO3A-fen was calculated to evaluate the advanced COX-2-binding affinity. COX-2 has active sites including a hydrophobic pocket, a secondary pocket, and the entrance of the active site (Figure 3a) [28,30]. Most COX-2 inhibitors can selectively bind to the secondary pocket and hydrophobic channel of COX-2 simultaneously [19,31].

In this study, the nonselective NSAID fenbufen was used in the targeting site of Gd-DO3A-fen. Fenbufen was inserted deep into the hydrophobic pocket of COX-2 (Figure 3b). The carboxyl group in the compound showed an ionic interaction with the terminal amino group of Arg 513 (OH-NH_2_ distance = 2.78 Å). In addition to ionic interactions, the aromatic rings of both compounds were involved in π–π stacking with Trp 387. The binding energy (Glide score) of fenbufen was −7.43 kcal/mol (Supplementary Table S3) [32].

Interestingly, Gd-DO3A-fen conjugated with the nonselective NSAID exhibited high binding energies with COX-2. Based on their corresponding Glide score (docking score), which approximates the ligand-binding free energy, Gd-DO3A-fen was found to be more potent than NSAID alone, which showed higher fitness values (Gd-DO3A-fen, −8.62; Supplementary Table S3). This result can be attributed to several factors. Firstly, the targeting site of Gd-DO3A-fen still bound strongly with Trp 387 even though a portion of the ligand moved into the solvent because of the short linker between the Gd complex and NSAID (Figure 3c). Secondly, in the case of Gd-DO3A-fen, the H-atom of the amide bond of the linker underwent H-bonding with the Asp 515 backbone (distance H-O = 2.62 Å). Finally, the solvent-exposed portion of the Gd complex interacted with a relatively polar domain near the COX-2 surface, such as Gln 192, which significantly contributed to strong binding. Gd-DO3A-fen does not generally possess well-known pharmacophores with tight binding sites or appropriately sized secondary pockets in its structure. Instead, it increases the binding affinity for COX-2 using a different binding site.

The COX-2-binding affinities (*K*_a_) of Gd-DO3A-fen and fenbufen were measured by UV–vis spectrophotometry. The intrinsic absorbance of each compound was decreased by the addition of COX-2. The binding constants of Gd-DO3A-fen and fenbufen to COX-2 were 6.68 × 10^6^ and 3.18 × 10^6^ M^−1^, respectively (Figure 3d,e). The COX-2-binding affinity of Gd-DO3A-fen was 2.1-fold higher than that of the traditional NSAID, which correlated with the in silico study.

### 2.4. Cytotoxicity

**Figure 4:**
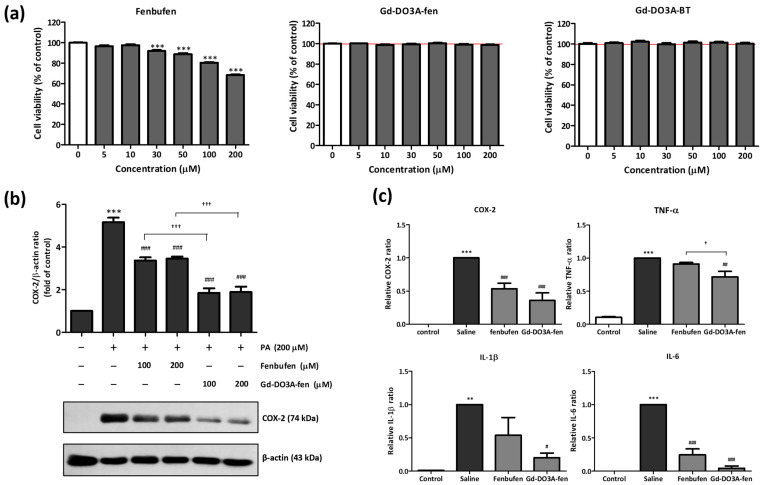
Antiinflammatory effect of drugs on PA-induced inflammation in C2C12 cells and in a turpentine oil-induced inflammation mouse model. (**a**) Effect of drugs on the viability of C2C12 cells (*** *p* < 0.001 vs. nontreated control; *n* = 3). (**b**) The protein levels of COX-2 analyzed in total cell lysates by Western blotting. The bar graph represents the average densitometry values relative to β-actin (*** *p* < 0.001 vs. nontreated control, ^###^ *p* < 0.001 vs. PA, ^†††^ *p* < 0.001 vs. fenbufen; *n* = 3). (**c**) Comparison of the mRNA expression levels of the proinflammatory enzyme and cytokines in the inflammation-induced mouse model injected with turpentine oil. The saline group was intravenously injected saline instead of drug in turpentine oil-induced model. The control group was injected with saline instead of turpentine oil. The bar graph represents the relative ratio of saline (** *p* < 0.05, *** *p* < 0.001 vs. nontreated control, ^#^ *p* < 0.01, ^##^ *p* < 0.05, ^###^ *p* < 0.001 vs. PA, ^†^ *p* < 0.01 vs. fenbufen; *n* = 3).

Our aim was to examine the intracellular protein regulation of Gd-DO3A-fen and NSAIDs in the in vitro study. For this purpose, it was necessary to first determine the concentration of Gd-DO3A-fen and NSAID to be used in the cells by performing an in vitro cytotoxicity assay using C2C12 cells treated with different concentrations of the drugs. Cytotoxicity was evaluated based on the cell viability relative to control cells. According to ISO 10993-5, in vitro cytotoxicity based on cell viability is rated as follows: >80% is noncytotoxic, 80–60% is weak, 60–40% is moderate, and <40% is strong cytotoxicity [33]. Figure 4a shows the cytotoxicity of the traditional NSAID, Gd-DO3A-fen, and Gd-DO3A-BT. Gd-DO3A-fen and Gd-DO3A-BT treatments had no significant effect on C2C12 cells at concentrations up to 200 μM. However, fenbufen treatment significantly decreased the viability of C2C12 cells, and only 68.32 ± 2.12% of control cells survived at 200 μM. We confirmed that fenbufen has weak cytotoxicity at 200 μM, but Gd-DO3A-fen has no toxicity up to 200 μM. These results illustrated that the toxicity of fenbufen was reduced by conjugation with the DO3A backbone. Gd-DO3A-fen could be used at a maximum concentration of 200 μM, which is noncytotoxic, in all our subsequent experiments.

### 2.5. Anti-Inflammatory Effect of Gd-DO3A-fen

To support the results of the in vivo MRI and docking simulation studies and to explore the interaction between COX-2 and Gd-DO3A-fen on a cellular level, we investigated the effect of Gd-DO3A-fen and fenbufen on the protein expression of COX-2 in C2C12 cells (Figure 4b). We found that palmitic acid (PA) stimulation, which triggers inflammation, expressed COX-2 in C2C12 cells (Figure S6). The COX-2 expression level was checked according to the treatment concentration of PA, and we found no significant difference between the PA concentrations of 200 and 500 μM. We performed an experiment in a follow-up study, with the PA concentration set to 200 μM. The result of Western blotting, in which the drugs were treated at concentrations of 100 and 200 μM, showed that the COX-2 expression elevated by PA was decreased by fenbufen treatment, but it was more significantly suppressed after Gd-DO3A-fen treatment. Thus, Gd-DO3A-fen reduced COX-2 expression more significantly than the traditional NSAID in the in vitro study.

We used a turpentine oil-induced inflammation animal model to compare the in vivo regulation of the mRNA expression of inflammatory mediators by Gd-DO3A-fen and fenbufen. Turpentine oil, similar to LPS or croton oil, can be used to induce innate immune responses and has been reported to induce sustained acute inflammatory responses [34]. In the turpentine oil-induced mouse model, inflammatory factors such as COX-2, TNF-α, IL-1β, and IL-6 were expressed in the saline-treated group. Gd-DO3A-fen clearly inhibited the mRNA expression levels of COX-2 as well as those of proinflammatory cytokines, such as TNF-α, IL-1β, and IL-6. However, the suppression of the inflammatory mediators COX-2 and IL-6 by fenbufen was statistically different (*p* < 0.001). Gd-DO3A-fen suppressed the expression of TNF-α more significantly than fenbufen (*p* < 0.01). These results suggested that the anti-inflammatory role of Gd-DO3A-fen is more effective than that of the original NSAID.

**Figure 5:**
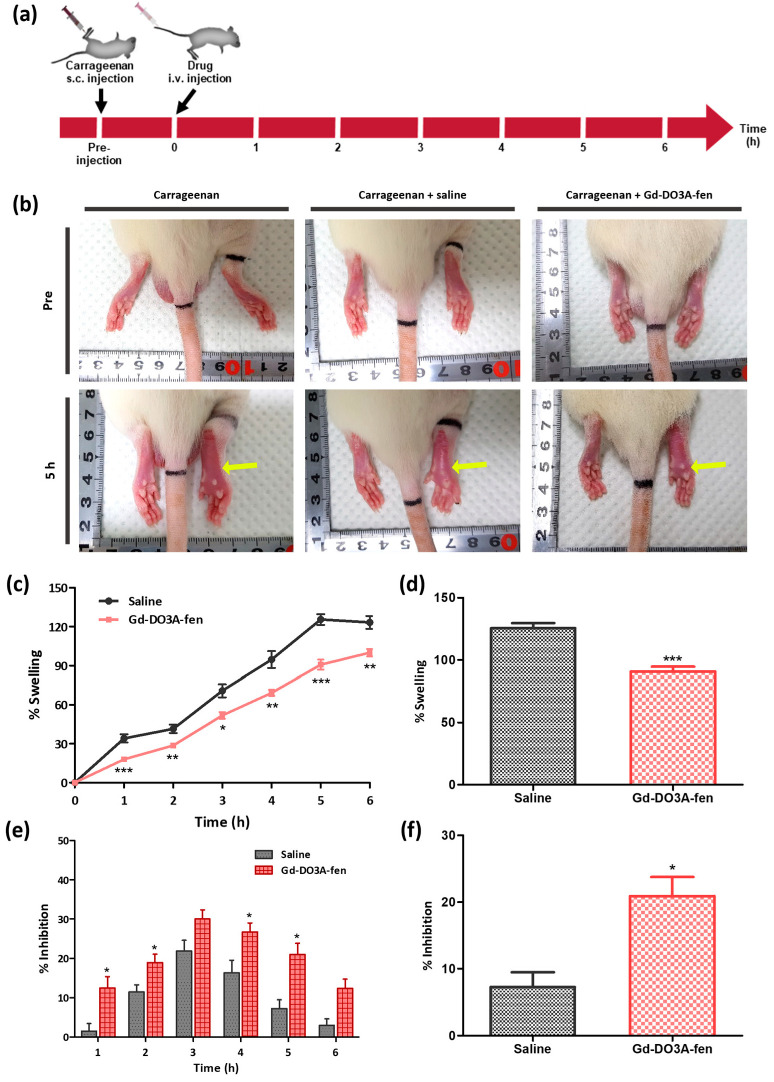
The inhibitory effect of Gd-DO3A-fen in the carrageenan-induced rat hind paw edema rat model. (**a**) The procedure for monitoring the anti-inflammatory effect of Gd-DO3A-fen on paw edema. The edema volume was measured before injection and at 1, 2, 3, 4, 5, and 6 h after the injection of carrageenan. (**b**) Images showing the rat paw before injection and at 5 h after the injection of carrageenan, respectively: carrageenan, carrageenan + saline, carrageenan + Gd-DO3A-fen. Yellow arrows indicate the carrageenan-induced rat paw. (**c**) Percentage swelling observed in the carrageenan-induced paw edema. (**d**) Comparison of % swelling of Gd-DO3A-fen group and that of saline group at 5 h after injection. (**e**) Percentage inhibition of paw edema by Gd-DO3A-fen and saline. (**f**) Comparison of % inhibition of Gd-DO3A-fen group and that of saline group at 5 h after injection. All values represent mean ± S.E.M. (*n* = 4 per group; *p < 0.05, **p < 0.01, ***p < 0.001 vs. saline group).

We also demonstrated the anti-inflammatory effect of Gd-DO3A-fen in the carrageenan-induced rat hind paw edema model, which is a classic model of inflammation (Figure 5a). This model is known to induce inflammatory parameters related to neutrophil activation, proinflammatory mediators, and inflammatory enzymes, such as COX-2 [35]. Subcutaneous injection of carrageenan to the paw stimulates an acute inflammatory process that results in its swelling [36,37]. In this study, we measured the volume of carrageenan-induced paw edema and analyzed the percentage of (%) swelling and inhibition of edema. When paw edema was maximal (5 h postinjection), reduced edema size was visually observed in the rat model treated with Gd-DO3A-fen (Figure 5b). The calculated values of % swelling and % inhibition of the two groups indicated that Gd-DO3A-fen suppressed the paw edema compared with the saline treatment group at most time points (Figure 5c,e). In particular, Gd-DO3A-fen reduced swelling by 72% and achieved 2.88-fold inhibition compared with the control treatment at 5 h postinjection (Figure 5d,f). These results clearly indicate that Gd-DO3A-fen is a highly potent anti-inflammatory agent in the in vivo study.

## 3. Discussion

Inflammation is a physiological problem commonly associated with human diseases, and there have been attempts to mitigate its effects through medicine [38]. Anti-inflammatory drugs, most commonly NSAIDs, continue to be developed and used. However, imaging technology is as important as novel anti-inflammatory medication for an early diagnosis of disease through the detection of inflammation [39]. In this study, we combined the two technologies to pursue the development of an anti-inflammatory agent conjugated with a Gd complex. For the first time, we have demonstrated that the anti-inflammatory Gd-DO3A-complex conjugated with fenbufen possesses diagnostic ability and also exerts therapeutic effects.

The Gd-DO3A-fen designed in this study demonstrated considerable effectiveness in terms of relaxivity and kinetic inertness compared with clinically used GBCAs. In particular, its strong, consistent, and prolonged enhancement in the inflamed tissue was remarkable, which is advantageous in the diagnosis of inflammatory diseases. This effect in the in vivo study is a consequence of its long circulation time, which was driven by the interaction between HSA found abundantly in the blood and Gd-DO3A-fen. The noncovalent interactions were generated by the lipophilic component represented by a conjugated aromatic moiety, such as fenbufen [40]. Jung et al. reported that the *K*_a_ values of **2b**, a Gd complex similar to Gd-DO3A-fen, is 1.07 × 10^2^ M^−1^, and that even MS-325, a blood pool CA with a very high binding affinity to HSA, has a *K*_a_ of 6.1 × 10^3^ M^−1^ [41]. Therefore, it is confirmed that the binding affinity of Gd-DO3A-fen to HSA cannot act competitively against COX-2 binding, as the *K*_a_ of Gd-DO3A-fen to COX-2 is 6.68 × 10^6^ M^−1^. The interaction between GBCA and HSA is limited to preventing immediate leakage into the interstitial space and prolonging the retention time in the blood.

We explored whether the structural modification of the NSAID with a Gd chelate renders sufficient alteration to the efficacy and mechanism of action of the NSAID to justify the inclusion of the modified drug as a new class of theragnostic. In generally, unlike nonselective NSAIDs, most selective COX-2 inhibitors have a building block, such as a diarylheterocycle or azide component, which possesses a methanesulfonic acid (SO_2_Me) COX-2 pharmacophore [31]. This can selectively bind to the secondary pocket and the hydrophobic pocket residues of COX-2 instead of COX-1 [19]. However, we found that the conjugation of a Gd chelate with the NSAID altered the COX-2-binding affinity inherently exhibited by the NSAID. In the case of Gd-DO3A-fen, the COX-2-binding affinity was improved without the presence of a pharmacophore building block. The Gd chelate, a bulky frame of Gd-DO3A-fen, may not be suitable for binding to the COX-2 pocket, but it instead creates a new binding site (e.g., at Gln 192) on the surface of COX-2, which interacts with a carboxylic acid moiety on the solvent-exposed part of the Gd chelate. Another result strengthening the case for its separate classification as a theragnostic agent is the unique structure–activity relationship strategy utilized by Gd-DO3A-fen to increase COX-2 inhibition. Amidation of carboxylic acid-containing NSAIDs renders them capable of binding to COX-2 [42,43,44]. Similar observations could be anticipated regarding amide bonding between the Gd chelate conjugated with fenbufen (Gd-DO3A-fen) and COX-2. However, according to in silico data, the amides of our Gd-DO3A-fen interact with Ser 353 and Asp 515 instead of the Tyr 355 and Glu 524 moieties of indomethacin amides [43]. Moreover, the molecular interactions between Gd-DO3A-fen and COX-2 suggest direct COX-2 inhibition. Finally, the in silico data suggested that Gd-DO3A-fen has a more potent direct COX-2 inhibition ability through enhanced COX-2-binding affinity than fenbufen, although further studies are required to confirm these in silico results. In addition, fenbufen is known to rapidly metabolize with active metabolites. In this study, although we speculate that fenbufen is less likely metabolized by conjugating with Gd-DO3A based on the kinetic stability measurement of Gd-DO3A-fen, future work is warranted to investigate whether Gd-DO3A-fen undergo metabolism in vivo.

In conclusion, we designed and synthesized an anti-inflammatory MRI agent and provided persuasive structural and functional reasons for its categorization as a new class of theragnostic agents. As an MRI agent, the *r*_1_ relaxivity of Gd-DO3A-fen was higher than that of the commercialized Gd-DO3A-BT that is clinically used. The kinetic inertness is well comparable with that of structurally stable macrocyclic MRI contrast agents. The targeting efficacy of inflammation sites by Gd-DO3A-fen was confirmed by an MRI in vivo study using a turpentine oil-induced animal model. Based on further experiments that demonstrated high COX-2-binding affinity by spectrophotometry and increased COX-2 inhibition effect in C2C12 cells, the MR enhancement in inflammatory lesions illustrated the targeting effect of the Gd complex on COX-2 overexpression in injured tissues.

We also evaluated the anti-inflammatory effect of Gd-DO3A-fen through in vivo experiments using a turpentine oil-induced thigh inflammation model and measured paw swelling in a carrageenan-induced edema model. Ryldene et al. reviewed the anti-inflammatory effect of compounds based on thiophene, one of the representative moieties of NSAIDs. Some introduced compounds modulated the gene expression of cytokines and/or inflammatory cytokines, such as TNF-α, IL-1β, and IL-6, in in vitro studies, and other compounds reduced the paw volume and inhibited the expression of COX-2 enzymes in a carrageenan-induced edema model [35]. Similarly, in our study, the anti-inflammatory activity of Gd-DO3A-fen was significantly assessed by measuring the size of edema suppressed in a carrageenan-induced paw edema model. Moreover, the mRNA expression levels of TNF-α, IL-1β, and IL-6, as well as that of COX-2, were reduced in a turpentine oil-induced thigh inflammation model. We found that Gd-DO3A-fen has an action mechanism related to the reduction of gene expression and/or inflammatory cytokines. Associating this with the result of the docking simulation between Gd-DO3A-fen and COX-2, which indicates the unique specific binding sites at Asp 515, Ser 353, and Gln 192 moieties, these data imply direct COX-2 inhibition by the Gd complex.

Overall, the good correlation between the in vitro, in silico, and in vivo assays demonstrates that Gd-DO3A-fen may be used as a potent form of anti-inflammatory agent, enabling inherent NSAIDs to significantly exert their therapeutic effect.

## 4. Materials and Methods

### 4.1. Materials

The reagents fenbufen, N,N’-dicyclohexylcarbodiimide (DCC), N-hydroxysuccinimide (NHS), and gadolinium (III) chloride hexahydrate (GdCl_3_∙6H_2_O) were purchased from Sigma Aldrich (St Louis, MO, USA). All other chemical reagents and solvents were obtained from Tokyo Chemical Industry Co., Ltd. (Tokyo, Japan) and Duksan Pure Chemical Co., Ltd. (Ansan, Republic of Korea). Tri-*tert*-butyl 2,2′,2″-(10-(2-((2-aminoethyl)amino)-2-oxoethyl)-1,4,7,10-tetraazacyclododecane-1,4,7-triyl)triacetate (DO3A-*^t^*Bu-NH_2_) was prepared according to the methods described in the literature [45]. λ-Carrageenan, PA, and other reagents were obtained from Sigma Aldrich (St Louis, MO, USA). Cell Counting Kit-8 (CCK-8) was purchased from Dojindo Laboratories (Kumamoto, Japan). Antibodies for COX-2 (Catalog No. 12282) were purchased from Cell Signaling Technology (Beverly, MA, USA), and β-actin (Catalog No. sc47778) was purchased from Santa Cruz Biotechnology (Santa Cruz, CA, USA).

### 4.2. Synthetic Procedures

#### 4.2.1. 2,5-Dioxopyrrolidin-1-yl 4-([1,1′-Biphenyl]-4-yl)-4-oxobutanoate (**1**)

Fenbufen (2.5 g, 9.83 mmol) dissolved in 40 mL of THF was added to NHS (2.7 g, 23.59 mmol). The mixture was stirred at room temperature for 30 min, and then N,N’-dicyclohexylcarbodiimide (2.43 g, 11.80 mmol) was dissolved in 30 mL of THF at 4 °C. The reaction mixture was stirred overnight. After completion of the reaction, the suspension was filtered, and the solvent was removed using a rotary evaporator. Open column chromatography (silica, DCM/MeOH, 99:1) was performed for purifying the crude product to afford **1** (1.65 g, 48%) as a white solid. ^1^H NMR (500 MHz, DMSO) δ 8.08 (d, J = 8.3 Hz, 2H), 7.84 (d, J = 8.0 Hz, 2H), 7.76 (d, J = 7.5 Hz, 2H), 7.52 (t, J = 7.6 Hz, 2H), 7.44 (t, J = 7.3 Hz, 1H), 3.48 (t, J = 6.2 Hz, 2H), 3.06 (t, J = 6.1 Hz, 2H), 2.82 (s, 4H). ^13^C NMR (126 MHz, DMSO) δ 196.68, 170.11, 168.70, 144.74, 138.82, 134.79, 129.08, 128.66, 128.41, 126.97, 126.91, 32.65, 25.42, 24.90. Anal. Calcd. for C_20_H_17_NO_5_: C, 68.37; H, 4.88; N, 3.99. Found: C, 68.35; H, 5.02; N, 4.08.

#### 4.2.2. Tri-Tert-Butyl 2,2′,2″-(10-(2-((2-(4-([1,1′-Biphenyl]-4-yl)-4-oxobutanamido)ethyl)amino)-2-oxoethyl)-1,4,7,10-tetraazacyclododecane-1,4,7-triyl)triacetate (**2**)

**1** (1.95 g, 5.55 mmol) dissolved in 20 mL of THF was mixed with DO3A-*^t^*Bu-NH_2_ (2.84 g, 4.63 mmol) in 20 mL of MeOH. After 48 h, the mixture was evaporated under reduced pressure. The crude product was dissolved in 100 mL of diethyl ether and extracted three times with 100 mL of deionized water. The organic layer was separated and dried by Na_2_SO_4_ and subsequently evaporated under reduced pressure. The crude product was roughly purified by open column chromatography (silica, DCM/MeOH, 96:4), which yielded **2** as a white solid. This product was used for the next step without further purification.

#### 4.2.3. 2,2′,2″-(10-(2-((2-(4-([1,1′-Biphenyl]-4-yl)-4-oxobutanamido)ethyl)amino)-2-oxoethyl)-1,4,7,10-tetraazacyclododecane-1,4,7-triyl)triacetic Acid (**3**)

**2** (1.68 g, 1.98 mmol) was dissolved in 30 mL of TFA/DCM (1:1) and cooled in an ice bath. The reaction mixture was stirred at room temperature for 24 h, and the solvent was removed using a rotary evaporator. The crude product dissolved in 10 mL of MeOH was precipitated into 200 mL of diethyl ether, and then the obtained solid was dried. Prep-HPLC was performed using a YMC-triart C18 column for purifying the crude product, which afforded **3** (1.56 g, 95%) as a white solid. The conditions were as follows: eluent A, 0.1% TFA in deionized water; eluent B, 0.1% TFA in MeOH; gradient, 5% B to 40% B in 3 min, 40% B to 80% B in 14 min, 80% B to 95% B in 5 min; flow rate, 12 mL/min. ^1^H NMR (500 MHz, MeOD) δ 8.11–8.07 (m, 2H), 7.79–7.74 (m, 2H), 7.69–7.66 (m, 2H), 7.49–7.45 (m, 2H), 7.42–7.38 (m, 1H), 3.87–3.80 (m, 4H), 3.73–3.68 (m, 2H), 3.40–3.28 (m, 16H), 3.20–3.08 (m, 8H), 2.69 (t, J = 6.5 Hz, 2H). ^13^C NMR (126 MHz, DMSO) δ 196.68, 170.11, 168.70, 144.74, 138.82, 134.79, 129.08, 128.66, 128.41, 126.97, 126.91, 32.65, 25.42, 24.90. Anal. Calcd. for C_34_H_46_N_6_O_9_∙2TFA∙H_2_O: C, 49.14; H, 5.43; N, 9.05. Found: C, 49.04; H, 5.62; N, 9.00. HR-FAB-MS (*m*/*z*): Calcd. for C_34_H_47_N_6_O_9_: 683.3405 [MH]^+^; found, 683.3408.

#### 4.2.4. Gd-DO3A-fen (**4**)

**3** (0.55 g, 0.806 mmol) dissolved in deionized water was adjusted to pH 3 using 1 M NaOH solution. Then, GdCl_3_∙6H_2_O (0.33 g, 0.886 mmol) solution was added dropwise, and 1 M NaOH solution was used to increase the pH level to 7. The reaction mixture was stirred at 55 °C for 18 h, after which the water was removed under reduced pressure. The crude product was purified by HPLC, which yielded **4** (0.38 g, 56%) as a white solid. The conditions were as follows: eluent A, 10 mM CH_3_CO_2_NH_4_ in deionized water; eluent B, 1 mM CH_3_CO_2_NH_4_ in ACN; gradient, 5% B to 45% B in 5 min, 45% B to 80% B in 15 min, and 80% B to 95% B in 5 min; flow rate, 12 mL/min. Anal. Calcd. for C_34_H_43_GdN_6_O_9_∙4H_2_O: C, 44.92; H, 5.66; N, 9.24. Found: C, 44.88; H, 5.37; N, 9.17.HR-FAB-MS (m/z): Calcd. for C_34_H_44_GdN_6_O_9_, 838.2411, [MH]^+^; found, 838.2409.

### 4.3. Transmetallation Kinetics Study

This experiment was designed as described previously [46]. It was based on an experiment originally designed to investigate the evolution of the water proton relaxation rate (*R*_1_^P^), in which PBS (pH 7.4) contains an equimolar Gd complex and zinc chloride. To 1 mL of the paramagnetic complex solution (1 mM), 10 μL of ZnCl_2_ solution (100 mM) was added. The mixtures were shaken, and samples were collected at 72 h. The *R*_1_ relaxation rate was defined as the reciprocal of *T*_1_ relaxation time. *R*_1_^P^(t)/*R*_1_^P^(0) was an index for estimating the extent of transmetalation. This study was conducted on a 3 T whole body system (Magnetom Tim Trio, Siemens, Munich, Germany) at room temperature (22–25 °C).

### 4.4. Relaxivity

This experiment was performed according to previous studies [47,48]. *T*_1_ measurements were performed using an inversion recovery method with a variable inversion time (TI) at 1.5 T (64 MHz) [46]. For *T*_1_ measurements, images were acquired at 35 different TIs ranging from 50 to 1750 ms. For *T*_2_ measurements, images were acquired with 28 different echo times (TE) ranging from 10 to 1300 ms using a CPMG (Carr–Purcell–Meiboon–Gill) pulse sequence. The relaxation times, *T*_1_ and *T*_2_, were calculated through a nonlinear least square fit using the SI of images and were expressed as the reciprocal of relaxation rates *R*_1_ and *R*_2_. Finally, the determined *R*_1_ and *R*_2_ were image-processed to obtain the map of relaxation rates. Those in a 0.67 mM solution of HSA were also obtained using the same protocol. Values are expressed as mean ± S. D. (*n* = 3).

### 4.5. Animals

Ethical regulations: all animal experiments were conducted according to the rules of the animal research committee of Kyungpook National University (Daegu, Republic of Korea). The animals were provided free access to food and water within an appropriate temperature of 21–23 °C and 50–60% relative humidity. Mice were anesthetized using 1.5% isoflurane in oxygen. For the preparation of the turpentine oil-induced inflammatory model, Institute of Cancer Research (ICR) mice (male, aged 6 weeks, weighing 22–30 g) were used in this study. We injected 60 µL of turpentine oil into the left thigh muscle of the mice and housed them for 3 days with monitoring [49]. MR scanning and drug administration were performed on day 4 after turpentine oil induction. The mRNA expression levels of COX-2, TNF-α, IL-1β, and IL-6 were measured using the inflamed tissue isolated on day 5. For the preparation of carrageenan-induced paw edema model, Sprague–Dawley rats (male, aged 6 weeks, weighing 170–180 g) were used. Acute paw edema was induced by subcutaneous injection of a 1% λ-carrageenan solution (100 µL) dissolved in 0.9% saline into the subplantar region of the right hind paw of the mouse [50]. All animal experiments were approved and performed according to the guidelines of the Institutional Animal Care and Use Committee of Kyungpook National University (Daegu, Republic of Korea; No. KNU-2019-0048, KNU-2019-0051).

### 4.6. In Vivo MR Image Acquisition and Processing

Inflammation-induced ICR mice (*n* = 3 per group) were scanned using a 1.5-T MR system (GE Healthcare, Milwaukee, WI, USA) with a home-made small animal radiofrequency coil, which was of the receiver type with an inner diameter of 50 mm. The spin echo parameters for *T*_1_-weighted images were as follows: repetition time (TR) = 300 ms; echo time (TE) = 13 ms; matrix size = 192 × 160; number of acquisitions (NEX) = 8; bandwidth = 15.63; scan time = 3 min 16 s. For coronal images of the whole body, slices of thickness 1.0 mm with a 10-cm field of view (FOV) and 0.5-mm spacing were used. For axial images of the inflammatory region, slices of thickness 1.0 mm with an 8-cm FOV and 0.5-mm spacing were used. Images were obtained continuously for 2 h and at 24 h postinjection of Gd-DO3A-fen, with Gd-DO3A-BT used for comparison. Image processing for gray scale and quantitative measurements with SI in the inflammatory region were performed using the ImageJ software (version 1.53a, National Institutes of Health, Bethesda, MD, USA), and the acquired data were represented as a CNR profile using OriginPro 8 (OriginLab, Northampton, MA, USA). The CNR was calculated using the formula CNR = (SNRpost − SNRpre), originally described in another report, where SNR is the signal-to-noise ratio [45]. Values are expressed as mean ± standard deviation (S.D.) The color-mapped images at maximum-enhanced time (2 h) and histograms of the inflammatory region were obtained using Python 3.6. All scripts were composed using the python library Matplotlib [51,52].

### 4.7. In Vivo Biodistribution Assay

Gd-DO3A-fen (0.1 mmol/kg) was administered intravenously through the tail vein to the inflammation-induced ICR mice (*n* = 4 per group) [53]. The mice were deeply anesthetized and sacrificed at 1.5, 6, and 24 h time points postinjection. The Gd concentration in tissue samples from various organ sites was measured (brain, heart, lung, liver, gallbladder, spleen, kidney, intestine, bladder, blood, and inflammatory tissue). Tissues in HNO_3_ (65%) were digested at 180 °C for 120 min. Clear samples diluted using 3% HNO_3_ solution were measured for Gd concentration by inductively coupled plasma atomic emission spectrometry (ICP-AES; Optima 7300DV, PerkinElmer, Waltham, MA, USA). The detection limit of this method was 0.01 ppm. The Gd accumulation was expressed as follows: percentage of Gd administration dose = measured Gd concentration per gram tissue/injected Gd amount. The data were plotted as mean values, and error bars were used to depict the probable experimental error.

### 4.8. Molecular Docking Study

The crystal structure of human COX-2 was acquired from the RCSB Protein Data Bank (https://www.rcsb.org; accessed on 15 January 2019; PDB ID was 5KIR). The struc-ture was processed using the Protein Preparation Wizard module (2018-1, Schrödinger, New York, NY, USA) for docking calcu-lations, and the three-dimensional structures of compounds were processed using Lig-Prep (LigPrep, 2018-1, Schrödinger, New York, NY, USA) of the Schrödinger suite of the software. Protein–ligand docking calculations were performed using Glide (Glide, 2018-1, Schrödinger, New York, NY, USA) (Grid-based Ligand Docking with Energetics) from the Schrödinger suite, which performs searches for favorable interactions between small mol-ecules and a receptor proteins. In Glide, molecules were described using an OPLS 2005 force field, and the SP (standard precision) mode of Glide was used, allowing for flexible ligand sampling. The molecular mechanics/generalized born surface area (MM/GBSA) feature of Prime was used for calculating the binding free energy (Prime, 2018-1, Schrödinger, New York, NY, USA) using the following equation:

Δ G_bind_ = E_complex_ − E_ligand_ − E_receptor_ (1)

### 4.9. COX-2-Binding Affinity Study

Solutions for 10 μM Gd-DO3A-Fen and 5 μM fenbufen were prepared in Tris-HCl buffer (pH 7.0) and HPLC-grade DMSO, respectively. A stock solution of 8.3 μL COX-2 (2.6 mg/mL) was diluted in 92 μL Tris-HCl buffer. Absorption values were read on a UV–vis spectrometer after each addition of 20 μL of COX-2 enzyme. The addition of COX-2 solution to GD-DO3A-fen and fenbufen was repeated until the absorbance intensity was saturated, and measurement was continued until the saturated intensity was repeated three times. Curve fitting to obtain *K*_a_ was performed using the following Benesi–Hildebrand equation:

1/(A_f_ − A_obs_) = 1/(A_f_ − A_fc_) + 1/*K*_a_(A_f_ − A_fc_)[L] (2)

and using saturation binding-one-site specific binding in GraphPad PRISM (GraghPad PRISM software Inc., Version 5.02, San Diego, CA, USA). In this equation, A_f_ is the absorbance of free compound, A_obs_ is the absorbance after the addition of COX-2 solution, A_fc_ is the absorbance at saturation, *K*_a_ is the binding constant, and [L] is the concentration of a compound.

### 4.10. In Vivo Study of Anti-Inflammatory Activity in the Carrageenan-Induced Paw Edema Rat Model

Acute paw edema was induced, and the volume of edema was measured using a LE7500 Digital Plethysmometer (Panlab, Harvard Apparatus, Cornellà, Spain) before and after the injection of carrageenan at several time points (before injection and after 1, 2, 3.5, 4.2, 5, and 6 h). Gd-DO3A-fen and saline were administered intravenously at 1 h after carrageenan injection. A volume of saline equivalent to the volume of Gd-DO3A-fen to be injected for treatment was administered as a control. The percentage of swelling was calculated as follows:

Percentage swelling = [(*V_t_* − *V_0_*)/*V_0_*) × 100], (3)

where *V_0_* is the right paw volume before the induction of inflammation, and *V_t_* is the paw edema volume at each time point after Gd-DO3A-fen treatment [36]. The percentage of inhibition was calculated using the following equation:

Percentage inhibition = [(*V_c_* − *V_t_*)/*V_c_* × 100], (4)

where *V_c_* is the paw edema volume after the induction of inflammation in the saline control [50]. The edema volume measurement was repeated four times per mouse, and the values were represented as mean ± standard error of the mean (S.E.M.) for the data replicates in each group. Gd-DO3A-fen values were compared with those obtained from the saline group by group analysis (*n* = 4 per group, * *p* < 0.05, ** *p* < 0.01, *** *p* < 0.001; other comparisons were not significant).

### 4.11. Cell Culture

The C2C12 mouse myoblasts used in this study were purchased from ATCC (ATCC^®^CRL-1772). C2C12 cells were cultured in Dulbecco’s modified Eagle medium (DMEM, WelGENE, Daegu, Republic of Korea) containing 10% (*v*/*v*) fetal bovine serum and 1% antibiotics–antimycotics (Gibco, Grand Island, NY, USA). Cells were incubated at 37 °C and 5% CO_2_ conditions. To induce the differentiation of C2C12 cells, myoblast differentiation into myotubes was induced by culturing the cells in DMEM containing 5% (*v*/*v*) horse serum (Gibco, Grand Island, NY, USA) and 1% antibiotics–antimycotics (Gibco, Grand Island, NY, USA) medium. After inoculating the undifferentiated cells in a culture dish, and after stabilization, they were transferred to a differentiation medium to induce differentiation. After 3 days, the differentiated cells were treated with drugs to perform analysis. The medium was replaced with fresh medium every 2 days.

### 4.12. Cell Viability Assay

Cell viability was evaluated to confirm the cytotoxicity of the drug using Cell Counting kit-8 (CCK-8, Dojindo Laboratories, Kumamoto, Japan) according to the manufacturer’s instructions. C2C12 cells (1 × 10^4^ cells/well) were seeded in 96-well plates and incubated until stabilization. The next day, the cells were switched to serum depletion medium, and the drug was treated at each concentration (0, 5, 10, 30, 50, 100, and 200 μM) for 22 h. Gd-DO3A-BT and fenbufen were used as positive controls. Next, CCK-8 solution was added to each well, and the plate was further incubated for 2 h. The absorbance at 450 nm wavelength was measured using a microplate reader (SpectraMax i3, Molecular Devices, CA, USA). All experiments were conducted independently in triplicate.

### 4.13. RNA Isolation and Real-Time Reverse Transcription-Polymerase Chain Reaction

Total RNA was isolated from the obtained mouse thigh muscle using Trizol reagent (Life Technologies, Rockville, MD, USA) according to the manufacturer’s instructions. Total RNA (1 μg) was converted into complementary DNA (cDNA) by reverse transcription using AccuPower CycleScript RT PreMix (Bioneer, Daejeon, Korea). According to the manufacturer’s protocol, real-time PCR was performed on Quantstudio 3 Realtime PCR system (Thermo Fisher Scientific, Waltham, MA, USA) and Power SYBR Green Premix (Cat no. 4367659, Thermo Fisher Scientific). The PCR amplification was controlled as follows: hold stage: one cycle of 10 min at 95 °C, PCR stage: 40 cycles of 15 s at 95 °C, and 40 s at 60 °C, melt curve stage: 15 s at 95 °C, 60 s at 60 °C, and 15 s at 95 °C [28]. Relative mRNA quantification was performed using the Quantstudio™ Design&Analysis software (Thermo Fisher Scientific, Waltham, MA, USA). Primer information and product size are as follows: COX-2: 99 bp, 5′-GAACCTGCAGTTTGCTGTGGG-3′ and 5′-TCGCACACTCTGTTGTGCTCC-3′; TNF-α: 105 bp, 5′-GGTTCTTGTCCCTTTCACTCA-3′ and 5′-CCTCTTCTGCCAGTTCCA-3′; IL-1β: 110 bp, 5′-CCTCACAAGCAGAGCACAA-3′ and 5′-AGAAACAGTCCAGCCCATAC-3′; IL-6: 134 bp, 5′-CTCTGGGAAATCGTGGAAAT-3′ and 5′-CCAGTTTGGTAGCATCCATC-3′; GAPDH: 85 bp, 5′-CTGCTCCTCCCTGTTCCA-3′ and 5′-CACACCGACCTTCACCAT-3′. GAPDH was used as a reference gene. All experiments were independently performed three times, and the average value was displayed on a graph.

### 4.14. Western Blot Analysis

For the analysis of protein expression changes, C2C12 cells (5 × 10^3^ cells/dish) were inoculated into a 60-mm culture dish. After inducing differentiation for 3 days, palmitate acid or/and drugs were added to the growth medium and incubated for 24 h. Cells were harvested, and protein extraction and Western blotting were performed as described in a previous study [54]. Membranes were incubated with the following diluted primary antibodies: COX-2 (1:2000, Cell Signaling Technologies, Beverly, MA, USA, Catalog No. 12282) and β-actin (1:1000, Santa Cruz Biotechnology, Santa Cruz, CA, USA, Catalog No. sc47778). Membranes were then incubated with horseradish peroxidase (HRP)-conjugated secondary antibody (1:5000, Cell Signaling Technologies) at room temperature for 1 h. Immunoreactive bands were detected on a Chemiluminescence Western Imaging System (Supernova-Q1800TM, Centronics, Daejeon, Republic of Korea). Band intensities were normalized to β-actin and measured using ImageJ (version 1.50i; National Institutes of Health, Bethesda, MD, USA).

### 4.15. Statistical Analysis

Results were reported as mean values, and error bars were used to express S.D., S.E.M., or probable experimental error depending on the data. All data were compared by group analysis using the independent *t*-test of the Mann–Whitney U test depending on data normality. All biological data were evaluated by one-way ANOVA with Tukey’s test and unpaired *t*-test. Statistical analyses were performed using GraphPad PRISM (GraphPad PRISM software Inc., Version 5.02, San Diego, CA, USA) and SPSS statistics version 23 (SPSS Inc., Chicago, IL, USA).

## Data Availability

Not applicable.

## Supplementary Materials

The following supporting information can be downloaded at: https://www.mdpi.com/article/10.3390/ijms24076870/s1.
