# Supplementary material for: Nonsteroidal Anti-Inflammatory Drug Conjugated with Gadolinium (III) Complex as an Anti-Inflammatory MRI Agent
Source: Int J Mol Sci. 2023 Apr 6;24(7):6870. doi: 10.3390/ijms24076870 (PMC10095586; doi:10.3390/ijms24076870)
Supplement: Supplementary file 1 [file ijms-24-06870-s001.zip › ijms-2301902-supplementary.docx]

**Supplementary information**

**
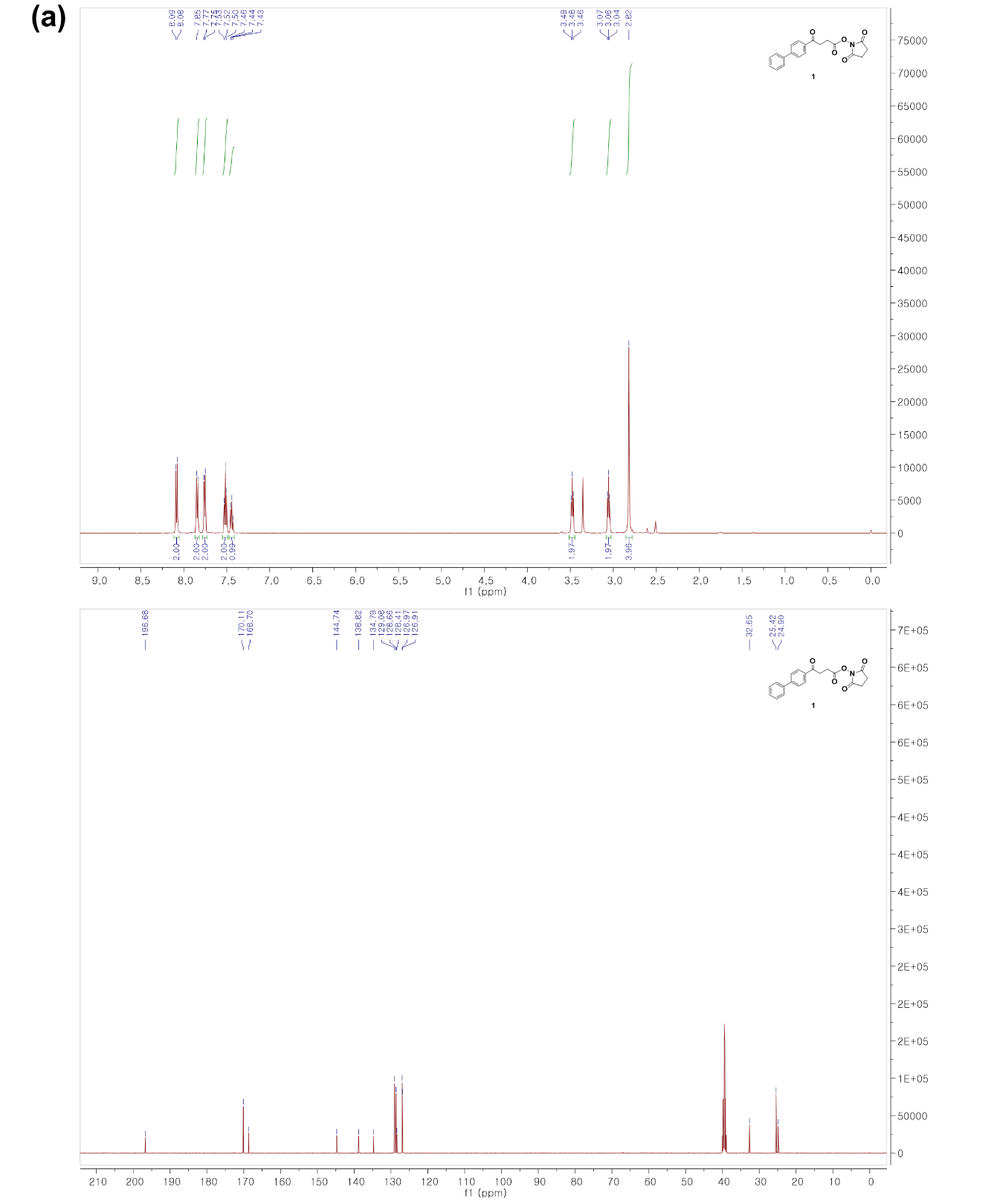

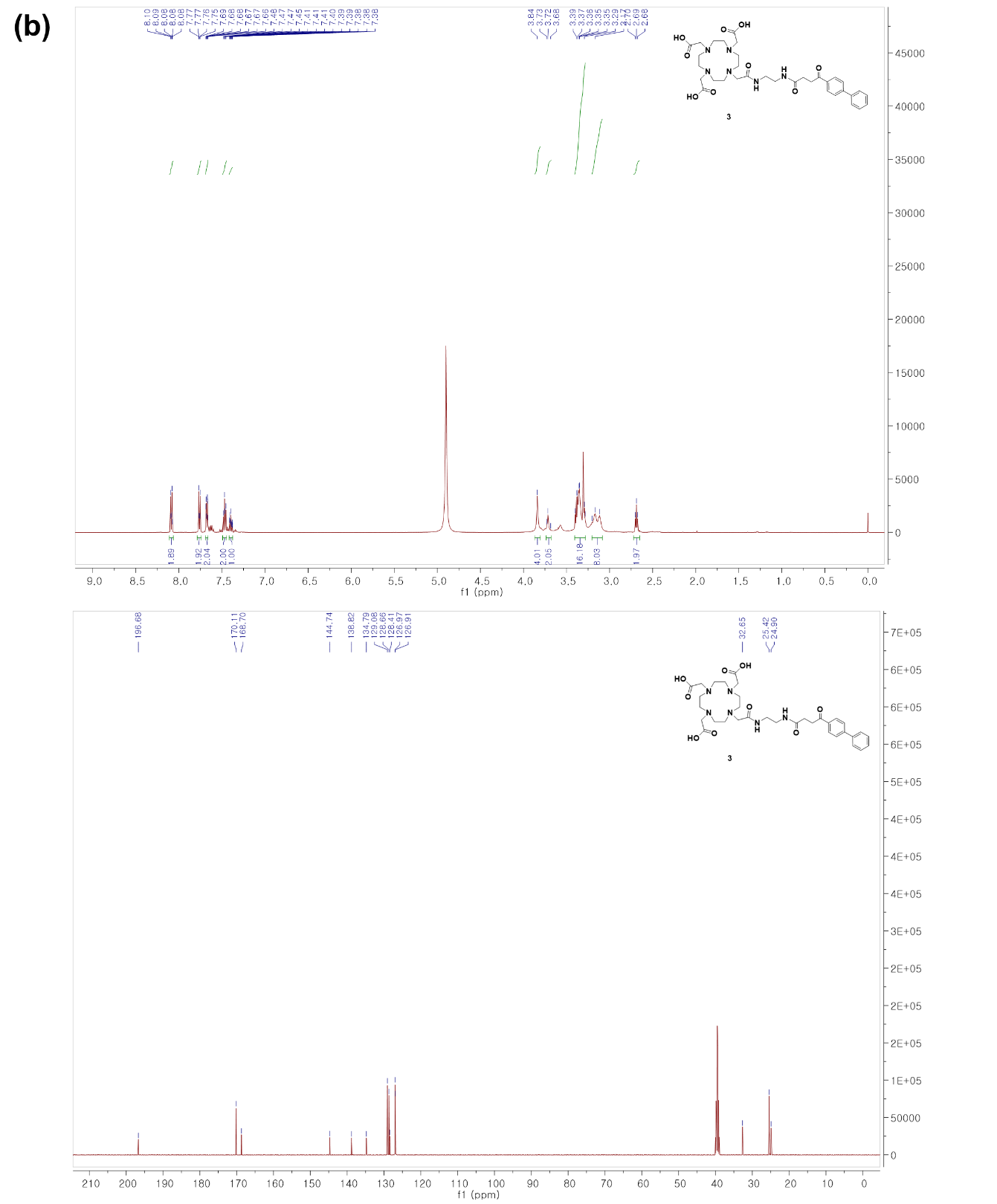
**

**Figure S1**. ^1^H and ^13^C NMR spectra of compound (a) **1**, (b) **3**.

**
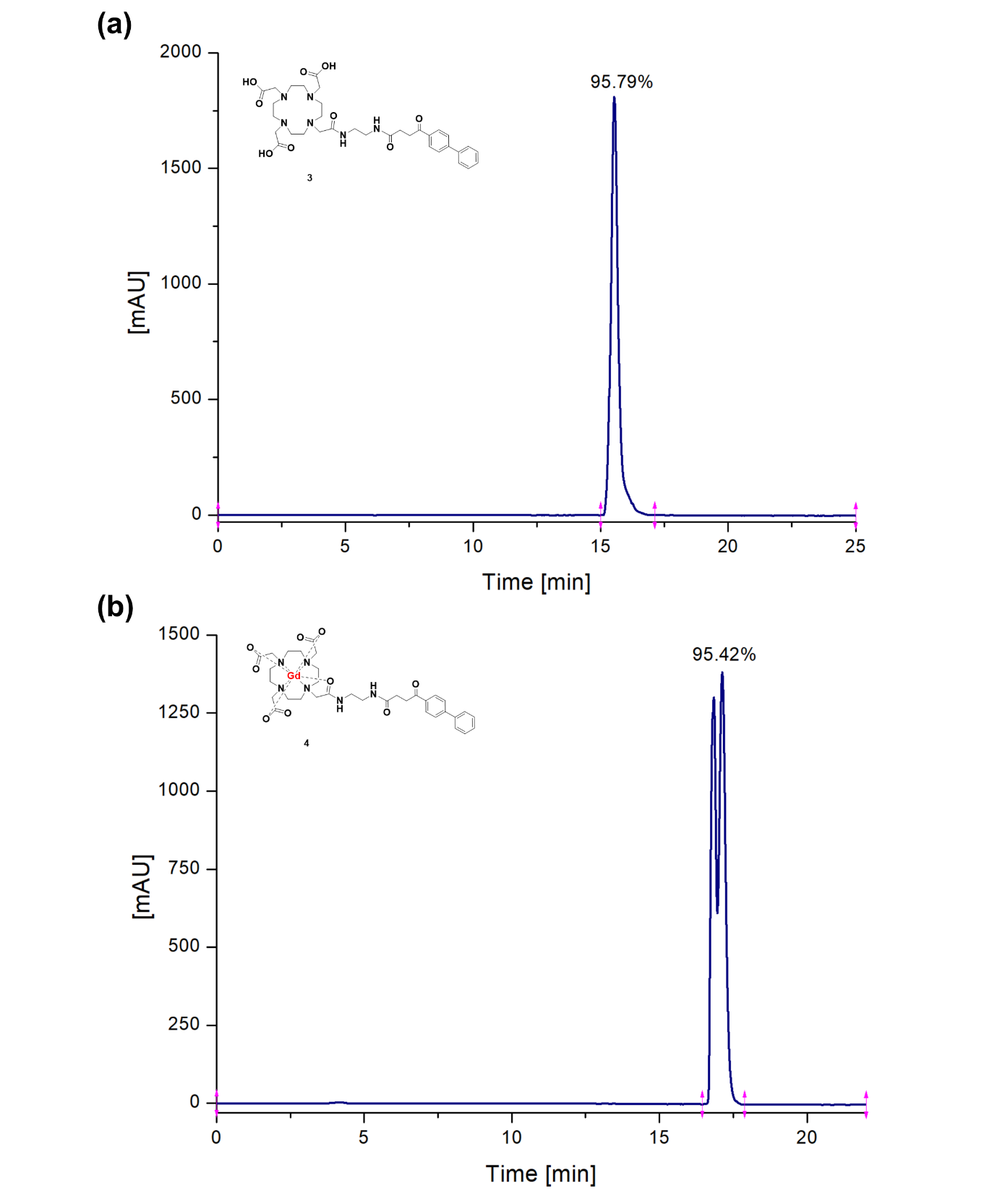
**

**Figure S2**. HPLC spectra of compound (a) **3,** (b) **4**.

**
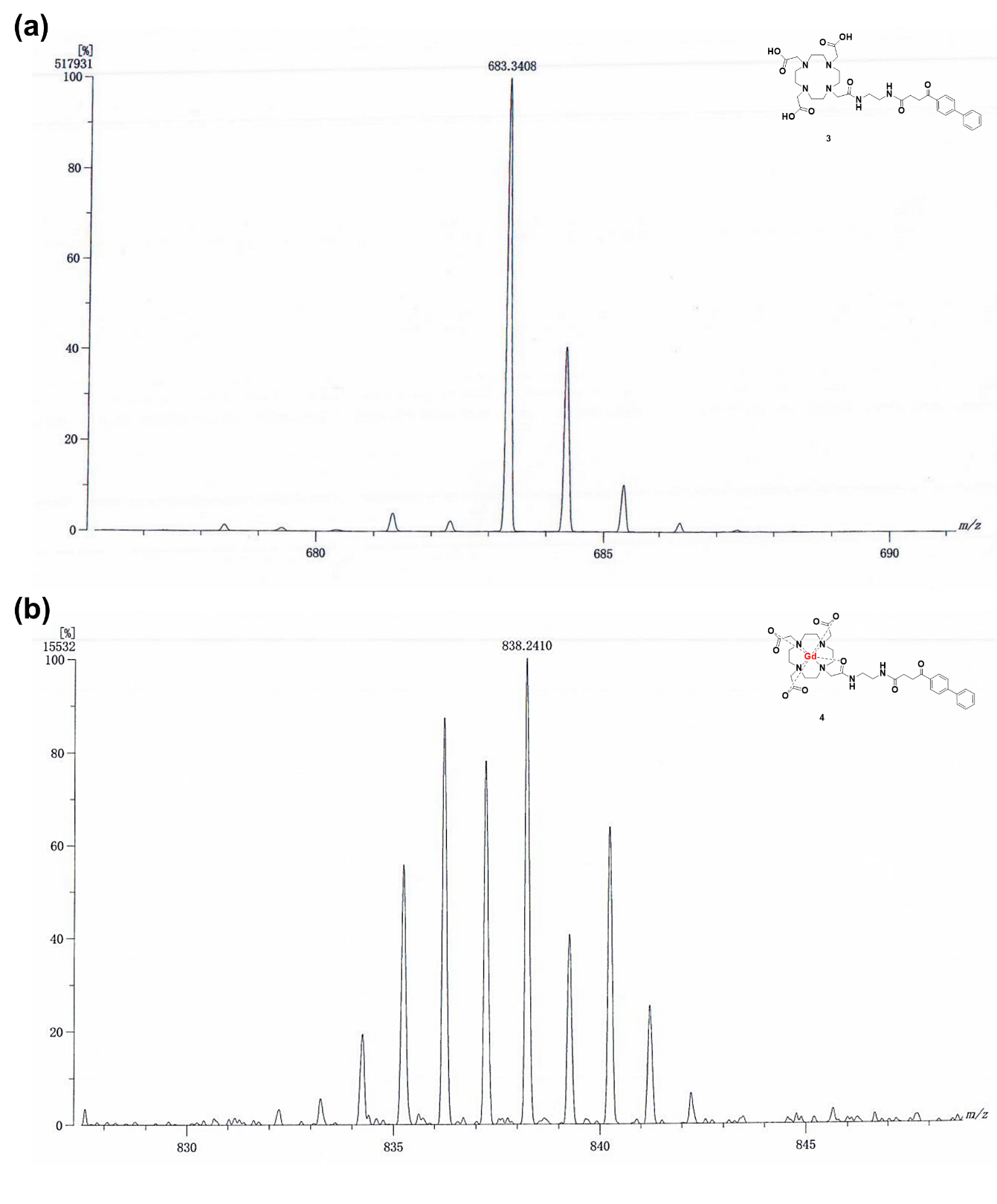
**

**Figure S3.** High resolution-FAB-MS spectra of (a) **3**, (b) **4**.

**Table S1.** Relaxivities of Gd-DO3A-fen and Gd-DO3A-BT in PBS and HSA solution were obtained using 1.5 T MRI as a slope of each *R*_1,2_ (versus Gd concentrations) with linear fitting graphs (adjusted *R^2^* > 0.994, *n* = 3). Octanol-water partition coefficients data of Gd-DO3A-fen and Gd-DO3A-BT.

| CAs | PBS | | HSA (0.67 mM) | Log *P*_oct/wat_ |
| --- | --- | --- | --- | --- |
|  | *r*_1_ (s^-1^mM^-1^) | *r*_2_ (s^-1^mM^-1^) | *r*_1_ (s^-1^mM^-1^) |  |
| **Gd-DO3A-fen** | 5.21$\pm$0.14 | 5.73$\pm$0.12 | 9.55$\pm$0.12 | -1.03 |
| **Gd-DO3A-BT** | 4.54$\pm$0.14 | 4.95$\pm$0.14 | 4.30$\pm$0.11 | -2.00 |

**
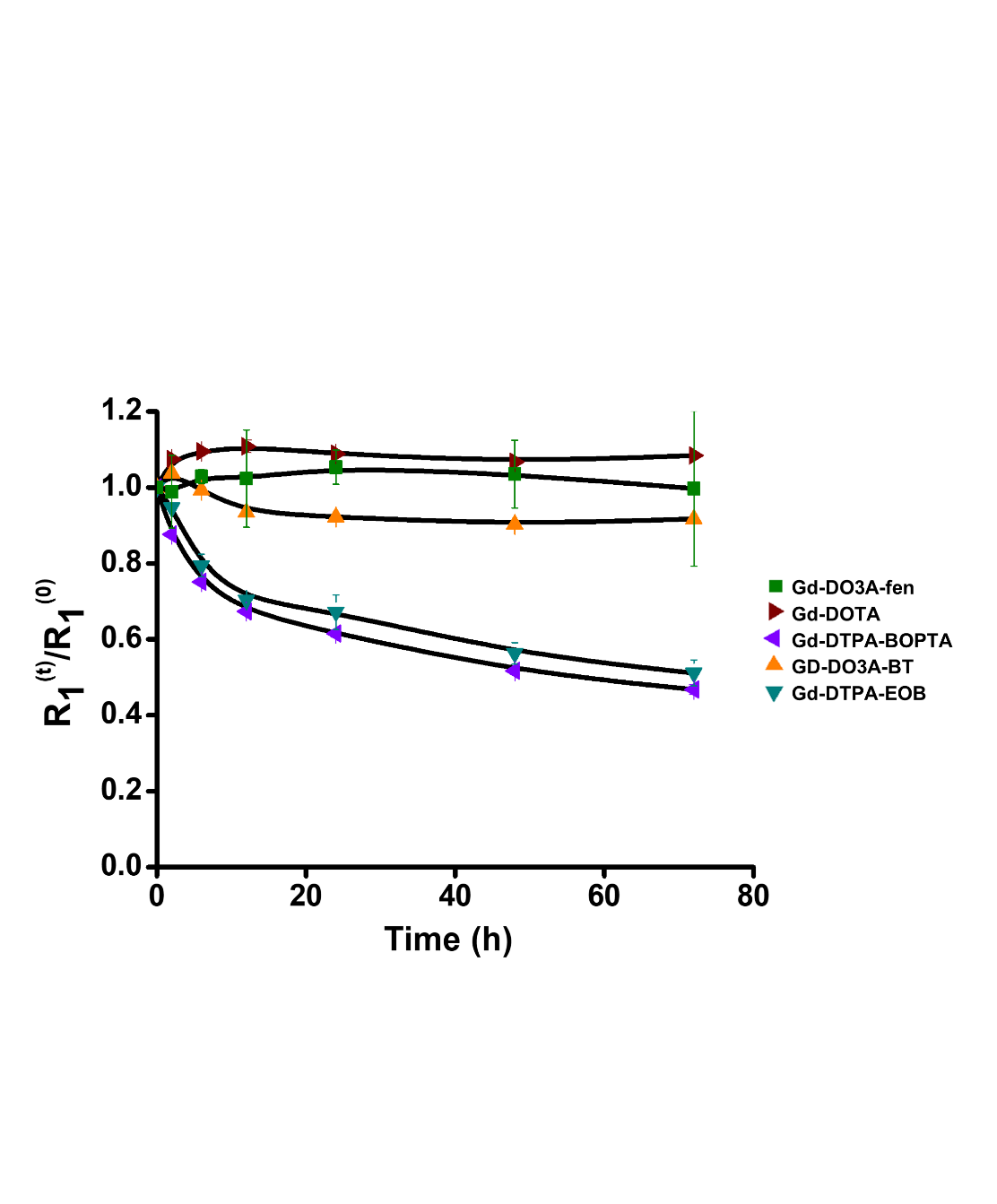
**

**Figure S4**. Kinetic stability was determined by measuring *R*_1_ change ratio against time in excess zinc chloride solution. In case of Gd-DO3A-fen, stability was approximately maintained at the initial *R*_1_ value.

**
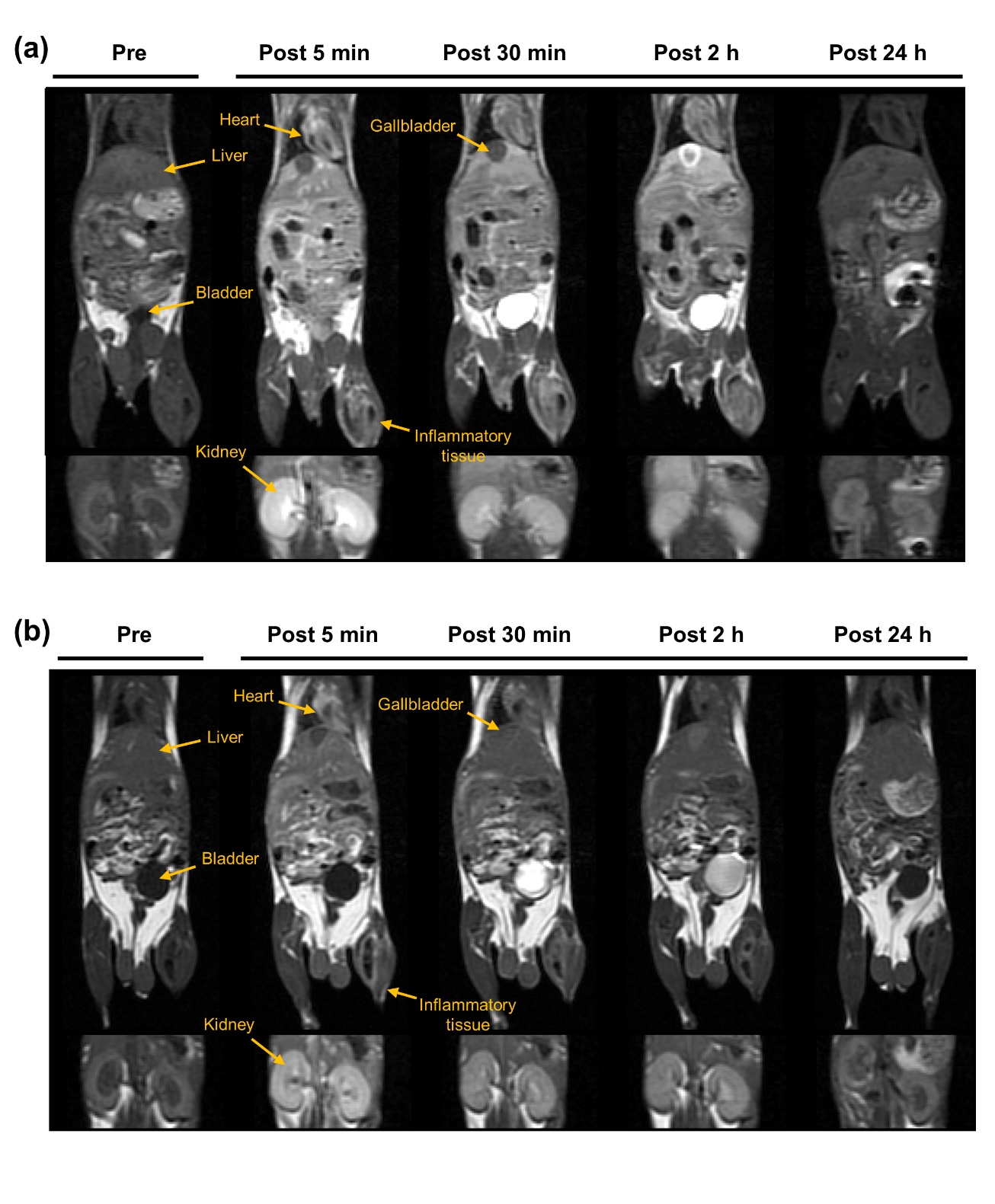
**

**Figure S5**. *In vivo* *T*_1_ weighted MR coronal images of mice obtained by tail vein injection of (a) Gd-DO3A-fen and (b) Gd-DO3A-BT (0.1 mmol /kg).

**Table S2.** CNR values of inflammation-induced mouse model obtained with Gd-DO3A-fen and Gd-DO3A-BT (Figure 2b).

| **Time (min)** | **Gd-DO3A-fen** | **Gd-DO3A-BT** |
| --- | --- | --- |
| 0 | 0 | 0 |
| 5 | 20.33 ± 1.27 | 16.72 ± 0.99 |
| 12 | 24.01 ± 2.11 | 13.31 ± 0.51 |
| 19 | 26.12 ± 2.09 | 12.65 ± 1.28 |
| 34 | 27.45 ± 1.66 | 10.65 ± 1.16 |
| 40 | 27.92 ± 1.40 | 9.27 ± 0.78 |
| 64 | 29.21 ± 0.50 | 7.89 ± 1.31 |
| 71 | 29.30 ± 0.63 | 6.39 ± 0.21 |
| 78 | 28.79 ± 1.09 | 5.97 ± 0.28 |
| 86 | 28.61 ± 0.39 | 5.76 ± 0.87 |
| 93 | 28.12 ± 0.05 | 5.34 ± 0.56 |
| 100 | 27.41 ± 0.92 | 4.49 ± 0.3 |
| 120 | 25.27 ± 0.91 | 3.65 ± 1.15 |
| 1440 | 0.47 ± 0.60 | 0.89 ± 0.79 |

|  | *Glide score (ΔG)* | *Glide evdw energy* |
| --- | --- | --- |
| Fenbufen | -7.431 | -26.981 |
| Gd-DO3A-fen | -8.623 | -49.762 |

**Table S3.** Glide score and calculated intermolecular energy (kcal/mol) of docking study. The glide score is an empirical scoring function that approximates the ligand binding free energy, including force field (electrostatic, van der Waals) contributions and terms rewarding or penalizing interactions known to influence ligand binding. The glide evdw energy intermolecular energy is sum of electrostatic and van der Waals energy.

**
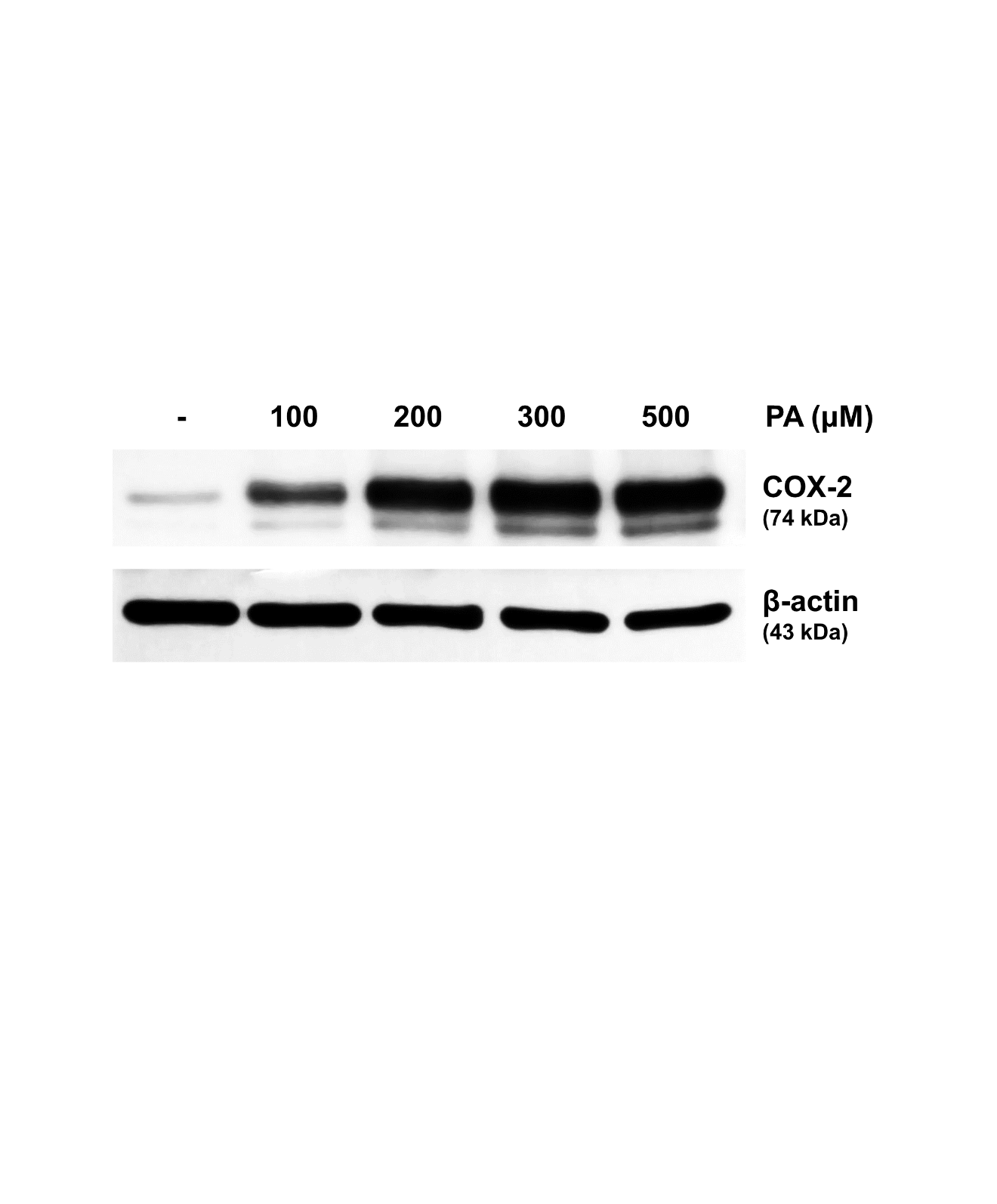
**

**Figure S6.** The expression pattern of COX-2 according to various concentration (100, 200, 300, and 500 μM) of PA.


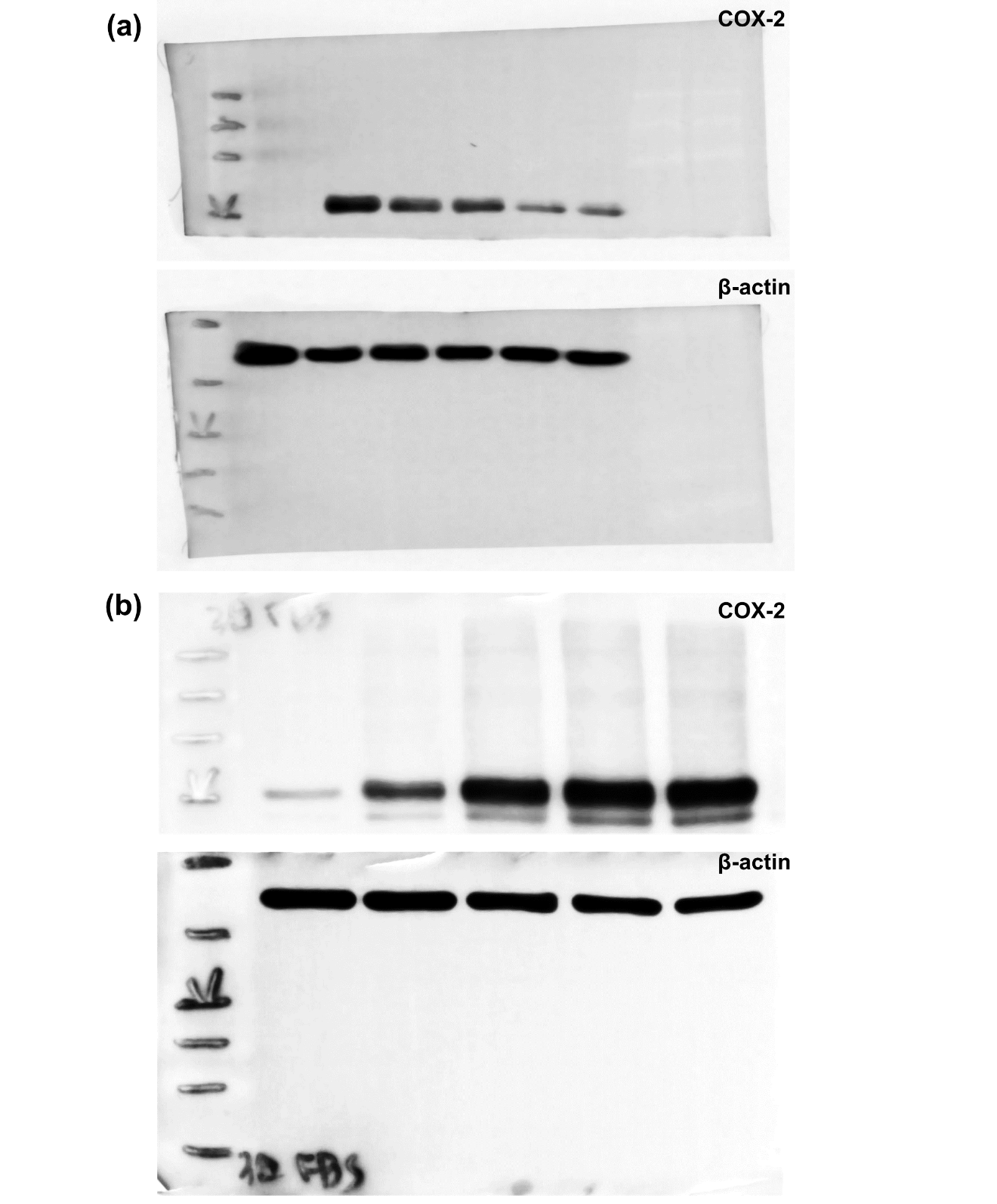


**Figure S7**. The source data of western blot analysis shown in (a) Figure 4b and (b) Figure S6.

**
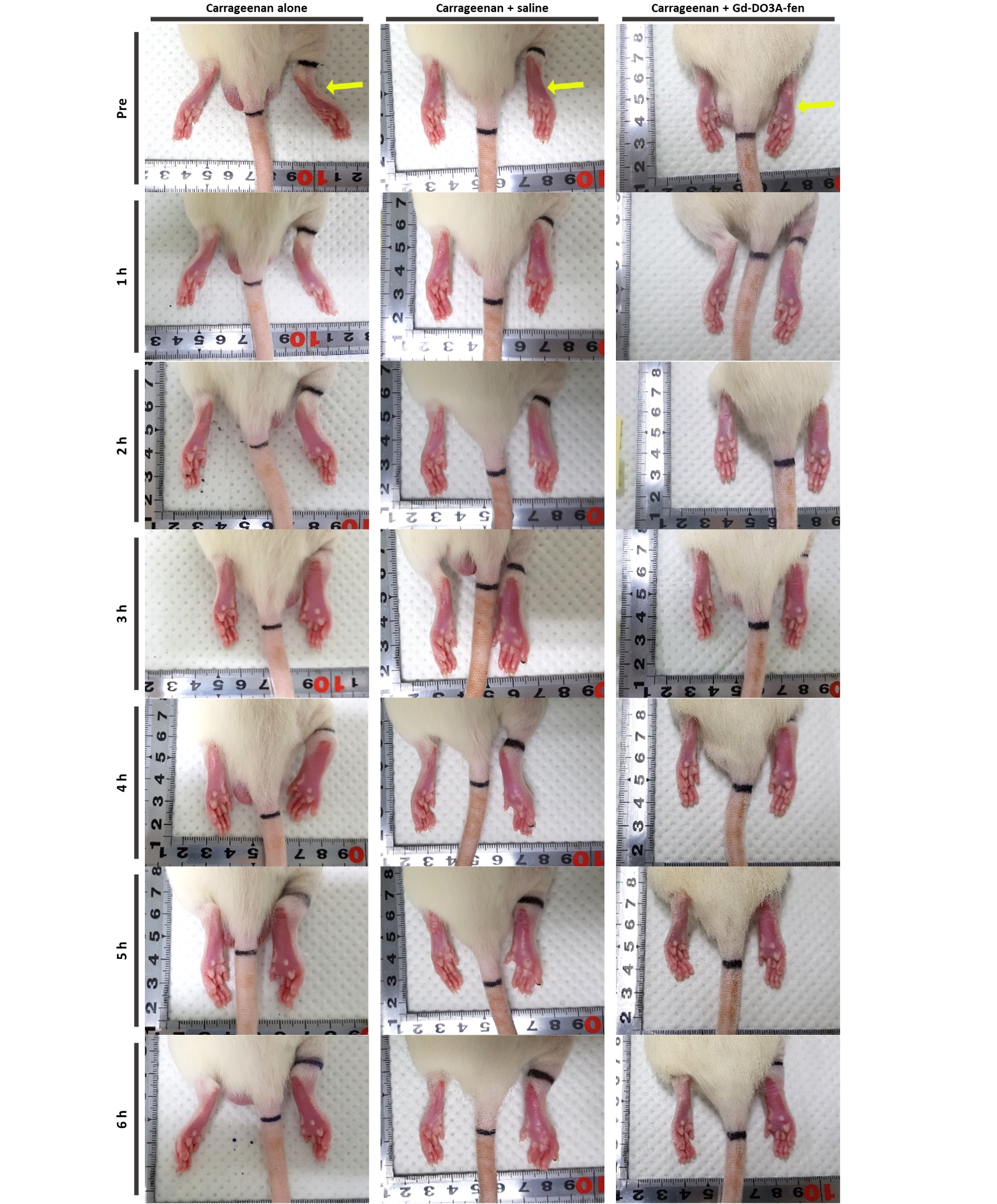
**

**Figure S8.** Representative images of rat paws of carrageenan, carrageenan + saline, and carrageenan + Gd-DO3A-fen groups. Each group shows pre-injection and 1, 2, 3, 4, 5, and 6 h after carrageenan injection (*n* = 4).

**Table S4.** (**a**) Percentage of swelling and (**b**) Percentage of inhibition of the paw edema for anti-inflammatory effect of Saline and Gd-DO3A-fen (Fig. 5c, e).

**(a)**

| **Time (h)** | **Saline** | **Gd-DO3A-fen** |
| --- | --- | --- |
| 1 h | 34.18 ± 3.16 | 18.04 ± 1.89 |
| 2 h | 41.52 ± 3.20 | 28.69 ± 1.86 |
| 3 h | 70.86 ± 5.04 | 51.93 ± 2.59 |
| 4 h | 94.70 ± 6.47 | 69.31 ± 2.31 |
| 5 h | 125.58 ± 4.12 | 90.80 ± 3.85 |
| 6 h | 123.38 ± 4.89 | 100.06 ± 2.86 |

**(b)**

| **Time (h)** | **Saline** | **Gd-DO3A-fen** |
| --- | --- | --- |
| 1 h | 1.50 ± 1.91 | 12.45 ± 2.88 |
| 2 h | 11.46 ± 1.78 | 18.89 ± 2.12 |
| 3 h | 21.85 ± 2.72 | 30.01 ± 2.31 |
| 4 h | 16.33 ± 3.17 | 26.69 ± 2.31 |
| 5 h | 7.26 ± 2.23 | 20.91 ± 2.88 |
| 6 h | 2.95 ± 1.68 | 12.38 ± 2.31 |
